# A Reminiscence of 1914: Christmas Morning with an Ambulance

**Published:** 1915-12-25

**Authors:** 


### A REMINISCENCE OF 1914.

### Christmas Morning with an Ambulance.

On Christmas Eve last year a spell of frost and bitter weather, which lasted several days, set in on the British Front in Flanders; and it served to accentuate the difficulties and discomforts of the troops to an extent which it is to be hoped they may be spared this year at Christmastide. It happened to be the duty of the writer to supervise for some days on each side of Christmas Day the removal of all the sick and wounded from one of the Divisions of the Expeditionary Force; and it is of Christmas Eve and Christmas morning that he preserves the most acute recollections during that tour of duty. The headquarters of the particular field ambulance to which he was attached was at a convent, situated some four miles or so, as the crow flies, from the trench line; and conveniently hidden from the German gunners by the configuration of the intervening country. This convent is very well organised and equipped as an orphan school. It is amply provided with modern buildings, laundries, electric-light plant, stables, farm buildings, dormitories, and all the accessories of a high-class institution, and can well bear comparison with British institutions of a similar kind. The society of nuns to whom it belongs surrendered cheerfully to the British about half the accommodation at their disposal, including their infirmary, their stables and outbuildings, cowsheds, and their principal hall, a roomy chamber which was converted into a vast ward capable on emergency of receiving more than a hundred patients. The most amicable relations subsisted between the good nuns and the British, both R.A.M.C. and patients.

### A Problem of Transport.

It is, however, not the working of the headquarters of the field ambulance, though that was interesting enough, that presents itself to the mind in connection with Christmas, 1914. About half-past five a bustle begins in that part of the courtyard where the ambulance waggons are kept, just outside the stables so as to be handy for harnessing up—motor-ambulances had not at that time been issued to field ambulances at the Front. Men appear leading large and very shaggy horses; by the light of occasional lanterns the men look even shaggier than the animals, for they are wearing goat-skin coats. With a certain amount of that genial profanity without which the British soldier is unaccustomed to conduct any military occupation, the horses are harnessed up, and the three waggons manœuvred into position on the exit road from the courtyard. A sergeant counts "heads," both of the waggon-drivers and orderlies and of the stretcher party which has been "warned" for duty with the evening collecting party, and when he has gathered in the last laggard reports "all present" to the officer. The latter, muffled almost to the eyes and wearing a multitude of woolly garments under his greatcoat and jacket, plunges out of the well-lit convent into the moonlight and confers for a few moments with the non-commissioned officer. That worthy agrees that the horses will have some difficulty in keeping their footing on the slippery, frozen roads; so to the dismay of the bearers they are informed that their usual privilege of riding out in the waggons is withdrawn for this occasion. The sergeant is instructed to see that this prohibition is observed, and the procession starts. The lamps of the waggons shed a feeble and flickering light on the road, but a waxing moon is of more help to the drivers and removes many of their difficulties. Soon after leaving the convent a stiff bit of gradient is encountered, which the first two waggons negotiate in good style. On reaching the level a halt is called, as the third waggon has not appeared. Presently it arrives, the horses blowing freely; but the sergeant opines that the cause of the delay has been poor driving, so after a brief breather for men and horses the order to proceed is given. On the hard, frosted road every hoof-beat rings musically, and one's own footsteps crunch so loudly that it seems as if the passage of the ambulance convoy ought to be audible for miles. There is, however, an exhilarating feeling in the crisp, dry, frost-bound air, so different from the damp, dank muggy cold which distinguishes Flanders when the temperature is slightly higher.

### In the Fire Zone.

A mile or more along the level, and we reach a point where an artillery ammunition column is replenishing the stock of shells of a battery hidden in an adjacent field. The road is too narrow for the ambulance waggons to pass the ammunition carts, so the leading driver is compelled to pull up. Hastening forward to see what is the obstruction, one meets a cheery and tactful sergeant-major, who assures one that he will be finished in a minute and clear out of our way. He is in such good spirits, and seems such a bustling, energetic sportsman, that he keeps the road blocked for nearly a quarter of an hour more without seriously annoying anyone. Advantage is taken of the delay to put out the waggon lamps and to pass the word for "no smoking"; for just over another small hill with a very steep gradient the road is (in daylight) in full view of the German gunners; and although they seldom fire at night, it is not worth taking any risks. Up this hill the horses have a certain amount of difficulty, even with the empty waggons; and visions of what may happen coming back are already beginning to worry the commander of the party. Then the road dips sharply downhill, and presently enters a small village, many of the houses in which are roofless or destroyed altogether. Were there daylight, there would be visible on both sides of the road leading down into this village the crater-like holes in the fields where high-explosive shells have landed, each crater full of muddy brown water, now coated thickly with ice. At a certain house in the village the waggons are halted. Here resides the medical officer of one of the battalions in the trenches a mile away, and from him an estimate of the sick and wounded from his battalion is obtained: some of the patients are in the house, others are still on the way. One waggon is left outside the house, with orders to turn round and face homewards, but not to load up until further orders. The other two waggons and the bulk of the bearers proceed through the village and along a road parallel with the trenches and about three-quarters of a mile behind them. There are three more collecting posts, and the first two of them visited are found to contain patients enough to fill one waggon, rather overfull certainly. One waggon is left at the second post in charge of the sergeant, with orders to take aboard the patients there, then to fill up with those from the post already passed, and then to return to the village and halt at the collecting post there. He is then to get the patients from the post in the village loaded on the empty waggon waiting there, and await the return of the third waggon. Meanwhile with a handful of bearers and the remaining waggon the last post is visited: it is the most exposed to fire, and stray bullets whistle over the waggon at frequent intervals along the road. The post is established in a dairy, and here a good many cases are waiting, including two or three seriously wounded and also an officer wounded in the foot an hour or so previously by a dropping bullet as he stood on the road just where the waggon is drawn up at the moment. When all are got on board the waggon is obviously overloaded, but a mental calculation shows that there are three or four vacant places on the other two waggons; and the road as far as the village is almost level. So back goes the procession to the village, passing the two collecting posts which the sergeant has been left to evacuate. Calling in at them for a moment to assure oneself that this has been done, the village is presently reached, where the other two waggons should be waiting to be picked up and to relieve the pressure in waggon number three. No waggons are there; inquiry reveals that they started homewards five minutes before, but no one seems to know why.

### Stuck Fast.

There is nothing for it but to follow them homewards, overloaded though we are. By this time it is long past ten o'clock; the frost is perceptibly harder and the half-moon is getting low in the sky. Out of the village the road is level, but a little way on a long hill is encountered—not anywhere quite so steep as the two short, stiff gradients encountered on the way out, but ending with a fairly steep pitch near the top, and nowhere contemptible. At the foot of the hill the precaution is taken to turn out of the waggon every man who is able to walk, so as to lighten the work for the horses. The roads are like glass, and presently the horses have to be rested. Another hundred yards and the waggon sticks again. A brief rest, and another effort is made, with everyone pushing at the wheels that is capable of helping. A few yards are gained, then another breather is necessary. This obviously bodes ill; no picks are available for roughing the road surface, but frozen soil is pulled out of the bank, broken into bits, and strewn on the road in front of the horses. Another great effort and the waggon reaches a stretch where the gradient is a little easier. With some difficulty this is passed, but the next bit defeats us entirely. The horses' feet are tied up in empty sacks to give them a better purchase; everyone except the helpless patients pushes with all his might; but to no purpose—the hill is too steep. Many are the maledictions upon the sergeant for disobeying orders and going off home. Had he stayed in the village, as he was told to, not only would the last load have been lighter, but the horses could have been "double-teamed" up the steep part of the hill, and the waggon would have gone up easily. There is only one thing to be done, and that is to return to the village and go home to the convent by another route, more than two miles longer, but possessed of easier gradients. Luckily the waggon is a Mark VI., and can be turned without undue difficulty on the narrow country road; had it been a Mark V., which some field ambulances in those days were saddled with, it would have been impossible to turn it on such a narrow, embanked piece of road.

### Home on Christmas Morning.

To cut a long story short, the convent was reached at 2 a.m. on Christmas morning, just as search parties for the missing waggon were being discussed; the first two waggons had been in for more than three hours. It transpired that a major of some combatant regiment had happened to pass through the village just as the loading of the two waggons was completed. This officer had been impressed with the fact that the waggons were nearly full of patients and were standing in the frost. As the result of his comments the medical officer at the collecting post in the village had, on his own responsibility, ordered the sergeant to take the waggons home at once. The result of this interference with the writer's carefully thought out arrangements was that two waggon-loads of men were saved five minutes on the road, but one overloaded waggon was three hours longer than it should have been. The sergeant, of course, should have explained that he had definite orders from the officer in charge of the convoy, and should have declined to accept an over-riding order from an officer of another unit. The breakdown had, at any rate, one good effect: it resulted in the loan of some motor ambulances for Christmas night, with the result that the collection was completed by 10.30, at which hour the writer was able to attack the remains of the Christmas fare placed before his colleagues at 7 o'clock! On another occasion, when collecting from the same area in very wild weather, a tree was blown down right across the road on the hill between the time of the outward and homeward trips. On this occasion, too, a return had to be made to the village and back to the convent by the long route. There were other memorable incidents on these trips; indeed, few of them were quite devoid of some distinguishing feature. But the Christmas Eve trip which extended into Christmas morning was, out of those in the writer's experience, that of which he has the most vivid recollection.

